# On the Base Composition of Transposable Elements

**DOI:** 10.3390/ijms23094755

**Published:** 2022-04-26

**Authors:** Stéphane Boissinot

**Affiliations:** Center for Genomics and Systems Biology, New York University Abu Dhabi, Saadiyat Island, Abu Dhabi P.O. Box 129188, United Arab Emirates; sb5272@nyu.edu

**Keywords:** transposable elements, GC content, base composition, codon bias

## Abstract

Transposable elements exhibit a base composition that is often different from the genomic average and from hosts’ genes. The most common compositional bias is towards Adenosine and Thymine, although this bias is not universal, and elements with drastically different base composition can coexist within the same genome. The AT-richness of transposable elements is apparently maladaptive because it results in poor transcription and sub-optimal translation of proteins encoded by the elements. The cause(s) of this unusual base composition remain unclear and have yet to be investigated. Here, I review what is known about the nucleotide content of transposable elements and how this content can affect the genome of their host as well as their own replication. The compositional bias of transposable elements could result from several non-exclusive processes including horizontal transfer, mutational bias, and selection. It appears that mutation alone cannot explain the high AT-content of transposons and that selection plays a major role in the evolution of the compositional bias. The reason why selection would favor a maladaptive nucleotide content remains however unexplained and is an area of investigation that clearly deserves attention.

## 1. Introduction

The base composition is one of the most fundamental properties of a genome or of a DNA sequence. Although all DNA sequences consist of 4 nucleotides, the relative proportion of Guanine/Cytosine (GC%) and Adenosine/Thymine (AT%) can differ considerably among organisms and among genomic regions. For instance, mammalian and avian genomes are highly heterogenous in base content, with gene-rich GC-rich compartments embedded in AT-rich intergenic regions, while reptiles, amphibians and fish are generally homogenous in base composition [1,2]. At a smaller scale, the GC% may vary among genes, among regions of a gene but also among codon positions. These differences in base composition can in turn affect a number of fundamental biological processes including transcription efficacy [3,4], the secondary structure of RNA molecules, translation efficacy and accuracy [5,6,7,8], the amino acid composition of proteins, and epigenetic modifications of DNA.

Transposable elements (TEs) are major components of genomes and have a profound impact on the size, structure, and function of their hosts’ genomes (Reviewed in [9]). Although most TE insertions are neutral or deleterious, TEs can also be a source of new genes or of regulatory motifs [9,10,11,12]. An aspect that has received little attention is the impact TEs can have on their host’s genome in terms of base composition. In many organisms, the base composition of TEs differ drastically from the genomic average and from hosts’ genes [13,14,15,16,17], to the point that the unusual base composition of TEs can be used to detect them in genomes [18]. This compositional bias, which is most commonly an AT-bias, may thus impact the structure and function of the genome in a number of ways. For instance, the accumulation of a type of TEs in specific genomic regions can potentially affect the GC genomic landscape, which in turn can affect other biological properties such as chromatin structure. Another interesting aspect is the effect the base composition of TEs can have on their own replication. In a number of organisms, the high AT% exhibited by TEs results in poor transcription and sub-optimal translation of TE-encoded proteins and thus seems maladaptive. Nevertheless, the AT-richness of TEs is widespread, and the persistence of such an unusual base composition across many categories of TEs remains a puzzle.

Here, I will review the state of our knowledge on the evolution of base composition in TEs, as well as the numerous questions that remain unanswered on this topic. After a short introduction on the biology of TEs, I will review what is known about their base composition, and in particular, I will emphasize that the high AT content observed for many TEs in many organisms is, in fact, not universal. I will then describe the consequences the unusual base composition of TEs may have on their hosts but also on their own replication. I will finally explore the evolutionary processes that are potentially driving the base composition of TEs towards nucleotide contents that appear, at first, maladaptive.

## 2. A Primer on Transposable Elements

**Figure 1:**
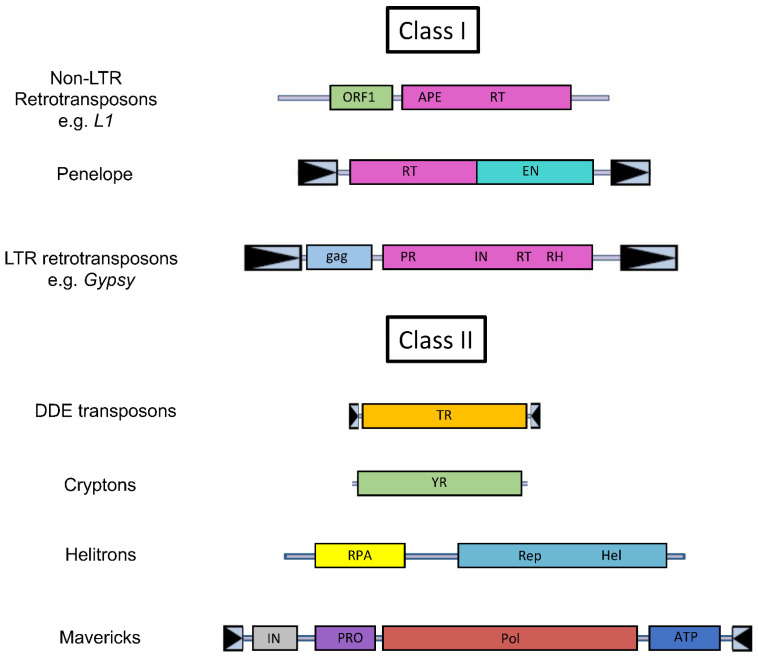
Schematic representation of the main categories of autonomous transposable elements. The elements are not drawn to scale. The following abbreviations are used: APE, apurinic endonuclease; RT, reverse transcriptase; ORF1, open-reading frame 1; EN, GIY-YIG endonuclease; gag, gag gene; PR, proteinase; IN, integrase; RH, RNase H domain; TR, transposase; YR, tyrosine recombinase; RPA, replication protein A; Rep, replication initiation domain; Hel, helicase; PRO, cysteine protease; Pol, protein-primed type B DNA polymerase; ATP, ATPase. The boxed arrows represent terminal repeats.

Transposable elements constitute a diverse group of sequences that have in common the ability to move from one location in the genome of their host to another location [19,20]. They are typically classified based on their mode of mobility [21,22]. Elements that move using an RNA intermediate are called class I elements, and those that do not are called class II (Figure 1). Each of these classes contains a myriad of subsets. Class I elements are further divided into LTR-retrotransposons, which are flanked by Long Terminal Repeats (LTRs) and include LTR-retrotransposons sensus stricto, endogenous retroviruses and *DIRS* elements, non-LTR retrotransposons (also called Long Interspersed Nuclear Elements or *LINEs*), and *Penelope* Elements. All these elements have in common the use of a reverse-transcriptase for their replication [23]. The replicative machinery of class I elements can also act on other transcripts and is responsible for the amplification of non-autonomous retroelements (such as Short INterspersed Elements or *SINEs*), which can far outnumber their autonomous progenitors [24,25,26]. Class II elements constitute a very disparate group of elements [27], which only have in common the fact that their replication does not require an RNA intermediate. They consist of four subgroups: DDE transposons that mobilized by a cut-and-paste mechanism mediated by a transposase, *Cryptons* that use a tyrosine recombinase for their transposition, *Helitrons* that use a rolling-circle mode of replication [28,29], and *Mavericks* that are mobilized by a self-synthetizing process mediated by a protein-primed polymerase B [30,31]. Class II elements can also mediate the transposition of non-autonomous copies, which can outnumber autonomous copies [32,33,34].

Class I and class II elements also differ by their long-term evolutionary dynamics within their host. Most *LINE*s in vertebrates are transmitted vertically over extended periods of evolutionary time and are thus long-term residents of these genomes. For instance, *L1* retrotransposons have persisted in the genome of mammals since the origin of this vertebrate class, and mammalian genomes contain a near complete record of the successive waves of *L1* amplification they have experienced since their origin [35,36]. The investigation of *L1* in mammals revealed that a very small number of lineages, often only one, persisted over long periods of time [35,37,38,39,40], which could reflect an arms race between *L1* and the repression machinery of the host [41,42]. In contrast, class II elements tend to invade the genome of their hosts by horizontal transfer, then amplify to large number but eventually get extinct [43,44,45]. Consequently, they rarely persist for long periods of time and are typically transient residents of genomes.

The number of TE copies and the diversity of elements differ considerably among genomes and depends on a number of parameters including the rate of transposition, the rate of fixation and the rate of DNA loss caused by deletions [46]. The rate of transposition depends on the number of progenitor copies and on the location of these progenitors in the genome (transcriptionally active vs. inactive genomic regions). The rate of transposition will also be affected by host-encoded repression processes. Since TE activity can be deleterious, a number of defense mechanisms have evolved to protect the integrity of the genome [42,47,48], DNA methylation being the best-known mechanism of defense against TE activity [49,50]. The rate of fixation will depend on the combined effect of purifying selection and genetic drift (reviewed in [46]) as well as linked selection [51]. Since the majority of new insertions is either deleterious or neutral, most of them are not expected to remain in the population and to be lost by chance (in the case of a neutral insertion) or to be eliminated by purifying selection (if the insertion is deleterious) [52,53,54,55]. However, in small populations, genetic drift can counteract the effect of selection and deleterious insertions can reach fixation [51,56,57,58,59]. Thus, one can expect that the overall rate of fixation and the accumulation of new copies will be higher in small populations than in large populations [60]. Finally, the number of TE derived sequence in a genome will depend on the rate of decay of these elements resulting from the rate of DNA loss by large deletions, which was shown to differ among organisms [33,61,62] and may be correlated to the number of copies (i.e., the accordion model of evolution) [63].

## 3. Variation in the Base Composition of Transposable Elements

Early analyses of base composition in transposable elements were focused on the composition of the ORFs, in the context of codon bias. Multiple codons encode for the same amino acid, yet there is often a bias in the use of synonymous codons (i.e., codons that encode for the same amino acid), where some codons are preferred over others. This is a common phenomenon in eukaryotes that is referred to as “codon bias” and has been the subject of extensive attention by evolutionary biologists [64]. Codon bias may result from selection in favor of codons that are optimal in terms of translation accuracy [5,8] or efficiency, which is supported by the observation that strongly expressed genes exhibit a stronger codon bias than weakly expressed genes [6,7,65,66]. Because of this relation between codon bias and expression, it has been proposed that the analysis of codon bias in TEs could inform on the nature of the interactions between TEs and their hosts [14]. Early studies in *Drosophila* found that the codon usage of TEs differed from the codon usage of the host, with a bias in favor of codons ending in A or T [16]. Further studies in model organisms (*Arabidopsis thaliana*, *Caenorhabditis elegans*, *Saccharomyces cerevisiae*, *Drosophila melanogaster* and *Homo sapiens*) revealed a general AT-richness of TE’s ORFs for both class I and class II elements compared with host genes [14] and a codon bias in TEs in favor of AT-ending codons, independently of the host. In most species, A-ending codons are preferred, except in the plants *A. thaliana* and *Oryza sativa*, where codons ending in T are preferred [17]. In general, the codon usage of TEs is different from the codon usage of host’s genes but tends to be similar to that of weakly expressed genes, at least in some species [14,17]. These observations suggest two things. First, a general mechanism, common to all TEs and independent of the host, may be responsible for the AT-richness of TE’s ORFs. Second, there is no tendency in TEs for codon optimization that would enhance the translation efficiency of the proteins they encode.

Although the general trend of an AT-richness and an AT-preference at the third position of codons seems to hold true for most TEs, more detailed analyses of a larger diversity of elements and the analysis of TEs in non-model organisms suggest a more nuanced and complex picture [15,67,68]. The analysis of base composition of non-LTR retrotransposons in vertebrates revealed large differences among clades of non-TR retrotransposons within the same genomes as well as large differences for the same clade among organisms [15]. For instance, in the lizard *Anolis carolinensis*, ORF2 (i.e., the ORF encoding for the reverse transcriptase) of elements belonging to the *L1* clade are enriched in AT (~67%) relative to host genes (~52% AT) while *L2* elements are GC-rich (~55% GC). In fish, *L1* and *L2* elements are AT-rich (~64% and ~58% AT, respectively) while elements of the *Rex1* clade (~52% GC) have a base composition close to the one of hosts genes (~50% AT). Although *L1* elements are universally AT-rich, there is a strong A bias on the positive strand in mammals and lizard (~41% and 43% A, respectively), a smaller bias in frogs (~33% A), and no bias in fish where A and T are equally represented. Interestingly, the base composition of the different clades is evolutionarily conserved and has persisted over long periods of evolutionary time within the same genome [15]. At the codon level, AT-rich codons are typically favored but there is no significant synonymous bias since the base frequency at the third position of codons fits the expectations given the overall nucleotide content of the sequences [15]. However, the codon usage of TEs tends to be closer to the codon usage of the host than expected given their base composition, which suggests a certain level of codon adaptation. Although TEs can be classified in AT-rich or GC-rich elements, some TEs show a highly unusual base composition. Such is the case of *L2* in the frog *Xenopus tropicalis* which is enriched in C (34%) and T (30%) on the positive strand [15]. These observations are not limited to vertebrates and similarly large differences in the GC% among class I elements were detected in the insect *Anopheles gambiae* [69].

Variation in base composition is not limited to class I elements. In a recent survey of GC content of TEs in fish, large variation in the base composition of class II elements was reported [68]. For example, class II elements in zebrafish are 36.9% GC while they are 44.1% GC in the pufferfish *Takifugu rubripes*. Interestingly, this study identified a positive correlation between the genomic GC content and the TE GC content suggesting an effect of the overall genomic environment on the base composition of TEs.

In some unicellular organisms, the pattern of base composition is drastically different. For instance, in the choanoflagellate *Salpingoeca rosetta* [70], all TEs exhibit a preference for GC-ending codons and for translationally optimal codons, thus suggesting selection for translational efficiency. Similarly, in the stramenopile genus *Phytophthora* [71], LTR retrotransposons show preference for GC-ending codons that mirrors host genes. Although additional analyses of unicellular eukaryotes will be necessary, this observation suggests some differences between unicellular and multicellular organisms, perhaps related to different effective population size between these categories [60,70].

Different regions of TEs can also differ considerably in base composition and even ORFs from the same elements can exhibit different base composition. This is exemplified in the mammalian *L1* retrotransposons which have 5′UTRs (57.2 GC%) and 3′UTRs (46.3% GC) that are richer in GC than the two ORFs (39.1% for ORF1 and 37.9% for ORF2) [13,15]. The GC-richness of the L1 promoter is consistent with the nucleotide content found around transcription initiation sites in vertebrates, which is in part due to the abundance of CpG dinucleotides [72]. The AT-richness of ORF1 in vertebrate *L1* is always higher than ORF2, which is consistent with the fact that much more ORF1 protein is produced than ORF2 protein [73,74]. The difference between ORFs is even more striking for elements of the *L2* clade. In lizard, *L2* elements have a GC rich ORF2 (55% GC) but an AT rich ORF1 (54% AT) [15].

Finally, the base composition of non-autonomous elements is extremely variable and is not necessarily related to the base composition of the autonomous elements responsible for their mobility. This is exemplified for *SINE* elements that are mobilized by *LINE* elements. The *Alu* element in primates, which is mobilized by the AT-rich *L1* element, is GC-rich (63.3% GC for *AluY*) while the *SINE* elements in mouse are either AT-rich (e.g., *B2* elements; 52.2% AT) or GC-rich (e.g., *B1* elements; 59.9% GC).

## 4. Consequences of the Unusual Base Composition of Transposable Elements

The unusual base composition of TEs has a number of consequences for the mobility of TEs but also for the genome of their hosts. First the AT-richness of TEs, which is prevalent in multicellular organisms, is suboptimal for the transposition process, both at the transcriptional and at the translational level. In mammalian *L1* elements, the A-richness of the positive strand results in poor transcription because A-rich L1 sequences constitute a poor substrate for transcription elongation (either because of a slower rate of elongation, stalling of the RNA polymerase complex or premature dissociation) and because of the presence of A-rich premature poly-adenylation signals that are causing early transcription termination [4,75,76]. It should be noted however that the number of canonical poly-adenylation signals differs among non-LTR retrotransposons and is not directly related to the AT-richness since the number of predicted poly-adenylation signals could vary more than two folds among elements with the same base composition [15]. Although experimental data are lacking for most TEs, it is likely that the AT richness of most TEs impedes their efficient transcription, but the prediction that elements devoid of AT bias exhibit a more efficient transcription remains to be tested. The second potentially negative consequence of a high AT content is at the translational level. The prevalence of AT-ending codons in most TEs makes codon usage of their ORFs poorly adapted for efficient translation, which is supported by the similarity between the codon usage of TEs and weakly expressed host genes [14]. From the point of view of TEs, their AT-richness may appear maladaptive since it negatively affects their transcription and the translation of their proteins.

The negative effect of the biased base composition of TEs is not limited to the TEs but can also impact the expression of host genes in a number of ways. For instance, an AT-rich element inserted within a host gene could decrease the transcription of the gene either by reducing the efficiency of transcription or by producing prematurely terminated transcripts [77,78]. This is one of the reasons AT-rich elements are rarely found in introns, and when they are, they tend to be oriented in the direction that is the least negative to gene expression [79,80,81]. This is exemplified in mammals where *L1* elements, which are AT-rich and have a strong A-bias on the positive strand, are extremely rare in introns, and the ones that have reached fixation are found in the opposite orientation to the host gene [79]. Another means TEs will affect the expression of host’s genes is via epigenetic regulation [82]. Repression by DNA methylation at CpG sites constitutes the main means of defense against transposon activity in many organisms [49,50]. Although AT-rich elements will by definition contain few CpG sites, elements that are enriched in GC can contain a number of CpG sites that will be the target of methylation. The repressive mark can spread to the flanking sequences of the transposons and occasionally affect the expression of neighboring genes. The fact that methylated TEs are on average found further away from genes than unmethylated TEs [83,84] and tend to be at lower frequency in populations [85,86] is consistent with a negative effect of TE repression on their neighboring genes.

The base composition of TEs will also affect the overall genomic composition as well as the structure and function of the genome. It is well known that the abundance of TEs is the main determinant of the haploid genome size and TE amplification can cause rapid genome expansion [87,88,89], yet the impact of TEs on the base composition of the host has been underappreciated. In a recent study in fish, a positive correlation between the genomic base composition and the base composition of TEs was found [68]. Since most TEs are AT-rich, small genomes that contain few TEs tend to have a higher GC content than genomes that have experienced large TE amplifications. This observation suggests that the GC content of TEs will drive the GC content of the genomes in which they amplify. This observation is not limited to fish, and the amplification of AT-rich TEs in fungi can cause rapid changes in the genomic base composition. This is exemplified in the genus *Leptosphaeria* where strains that have experienced TE amplification have genomes with a lower GC content (45% GC) than strains that have not (51% GC) [90].

The GC content can differ among genomic regions (e.g., in birds, mammals and gars) or can be relatively homogenous (e.g., reptiles, amphibians and teleost fish) [1,2,91,92]. The cause of GC heterogeneity in birds and mammals has been the subject of extensive research. It is believed that the main driver of base content heterogeneity is a process called GC-biased gene conversion [93], which causes a fixation bias of G:C alleles over A:T alleles by a recombination-dependent process. Thus, regions of high recombination tend to be GC-rich while regions of low recombination tend to be GC-poor. An aspect that has received little attention is the contribution of TEs to GC heterogeneity. In mammals, the differential accumulation of TEs that differ in base composition contributes to the GC heterogeneity of the genome. AT-rich *L1* retrotransposons accumulate in regions of low recombination, presumably because they are eliminated by purifying selection from high recombining regions due to their ability to mediate ectopic recombination [54,94]. They will thus contribute to the higher AT content of regions of low recombination. In contrast, their non-autonomous counterpart, the *Alu* SINE, is GC-rich and tends to accumulate in genic regions with a high recombination rate [79], thus contributing to the evolution of these GC-rich genomic compartments. Although TEs certainly have an effect on regional base composition, this effect remains to be quantified and the recent development of tools that jointly analyze base composition and TE distribution will contribute to solving this gap in our knowledge [95].

## 5. Why Do Transposable Elements Have Such Unusual Base Composition?

Two main questions emerge from the analyses of the base composition of TEs. First, why do some TEs exhibit a nucleotide content that is so different from the genome average or from host genes? And second, why do some TEs from the same genome have drastically different composition? The unusual base composition of TEs could result from a number of non-exclusive factors that fall into three broad categories: horizontal transfer, mutational bias, and selective pressure.

TEs that have recently invaded a genome by horizontal transfer will exhibit a base composition that does not reflect processes which have taken place within the genome they occupy. In this case, we do not expect those TEs to show evidence of adaptation to the base composition of their new host. For this reason, a bias in the codon usage of TEs (which is related to the base composition) has been used as evidence of horizontal transfer [96,97,98,99], although many vertically transmitted TEs exhibit a similar bias [15,100,101]. Horizontal transfer can also explain differences in base composition between elements within the same genome. For instance, the genome of the medaka fish *Oryzias latipes* contains three families of *RTE* retrotransposons that differ substantially in base composition, but since *RTE* is prone to horizontal transfer [102,103], it is likely that these differences are caused by the independent transfer of *RTE*s from different sources [15].

The previous explanation does not apply to elements that are strictly (or mostly) vertically inherited. Many elements have persisted in genomes for very long periods of evolutionary time and have thus had time to evolve within the context of their host, and in this case, the evolution of their nucleotide content can be affected by mutational bias and/or selective pressure. Analyses of the pattern of mutation of recent copies of non-LTR retrotransposons, which are in majority AT-rich, revealed that mutations from C to T and G to A are the most abundant ones and that this mutational bias affects all elements, independently of their clade or base composition [15]. Although the overall mutational bias towards A and T is consistent with the general AT-richness of TEs, it fails to explain the strand bias observed for some elements such as *L1*. The cause of this mutational bias remains unclear but could result from a number of processes. The use of an error-prone reverse transcriptase by class I elements is unlikely to play a major role because the misincorporation of dATP by reverse transcriptase is exceedingly rare ([104], although this has only been tested on retroviral reverse transcriptase) and because some class I elements, like *L2*, use a reverse transcriptase for their transposition, yet they can have high GC content. In addition, this does not explain the high AT% of class II elements, which do not rely on a reverse transcriptase for their transposition. Another possibility comes from the action of DNA editing enzymes of the APOBEC family which are part of the defense system against viruses and retrotransposons. APOBEC3 proteins cause G to A mutations on the positive strand and could thus contribute to the A richness of some *LINE*s [105], although the signature of such editing was detected in a very small fraction of *L1* elements in humans. Interestingly, it was shown that APOBEC affects differently the AT-rich *L1* and the GC-rich *L2* elements in *Anolis carolinensis*; *L1* exhibited a signature of APOBEC editing while *L2* did not show any [106], a pattern consistent with the different base composition of these two clades of *LINE*s. More research is needed to assess the effect of editing on a broader range of organisms and to quantify the impact of APOBEC enzymes on base composition in a variety of contexts. Finally, the genomic environment in which elements are inserted could affect the type of mutations they experience. GC-biased gene conversion will affect differently elements inserted in regions with different recombination rate. Elements that accumulate in low recombining regions would be less subject to GC-biased gene conversion than elements that reside in regions of high recombination and will thus diverge in terms of nucleotide content. This process could be exacerbated by TE-specific insertion bias in favor of genomic regions with high or low recombination rate [107,108,109]. This hypothesis could be tested by comparing the mutation spectrum of TEs residing in different genomic compartments. It should be noted that the bias towards AT is not general, and for instance, a mutation bias from AT to GC was detected in the choanoflagelate *Salpingoeca rosetta* [70]. Interestingly, TEs in this species do not exhibit the AT-richness found in other organisms.

Another observation suggestive of a mutation-driven evolution of base composition comes from the correlation between the GC% of TEs and the GC% of non-repeated genomic DNA in fish [68]. This positive correlation suggests that the genomic context could be having an effect on the base composition of TEs, but it is unclear how the genomic GC% drives the GC% of TEs. A possibility is that, for their replication, TEs have to use the pool of nucleotides available in the genome of their host, which thus constrains the base composition of TEs. Consequently, it is plausible that the composition of the pool of nucleotides will drive the evolution of the base composition of the TEs closer to the genomic average. This hypothesis remains to be tested, and the variation in base composition reported in teleost fish suggests that this group constitutes a good model.

A strictly neutralist mutational process is however unlikely to fully account for the unusual base composition of TEs, and a number of observations suggest that selection acts on base composition. First, in vertically transmitted TEs, such as *LINE*s, the base composition remains constant over long periods of evolutionary time suggesting selective pressure or functional constraint on elements [13,15]. This is exemplified in mammals where the *L1* retrotransposon has maintained its AT-richness and an A bias on the positive strand since the origin of this vertebrate class. Second, elements can differ in base composition within the same genome (e.g., *L1* and *L2* elements in *Anolis carolinensis*), although they are experiencing a similar pattern of mutation [15]. Third, different regions of the same element can exhibit a drastically different base composition. This is exemplified by the difference in base composition between the first and second ORFs of *L1* and *L2* in mammals and lizard [13,15]. Fourth, some TEs have retained a GC-rich or CT-rich content despite a mutational pressure towards A and T [15]. Fifth, in some species the codon usage is more similar to the host than expected given the base composition of elements, indicating a certain level of codon adaptation.

It is relatively easy to explain the base composition of TEs that exhibit GC% similar to their host’s genes and those cases exemplify adaptation of TEs to their host [70]. It is much harder to provide a selectionist explanation for the apparently maladaptive base composition reported in many TEs. The first possibility is that the unusual nucleotide composition of TEs is not detrimental but is in fact adaptive and is responsible for the fine-tuning of TEs’ transcription and translation. In this context, it is plausible that the high AT content is a mechanism of self-regulation reflecting a trade-off between the efficiency of transposition and the negative impact of transposition on the host [75]. For instance, selection could favor an inefficient transcription of TEs because an element that would be transcribed too efficiently could transpose at a level that would be detrimental to the host [76]. Selection at the transcriptional level seems more likely than selection at the translational level since the AT-richness is often observed at the three codon positions [15]. It is not to say that selection acts only to reduce the efficacy of transposition. In some species, the codon usage tends to be more similar to the host than expected given the composition of the element, which is evidence in favor of adaptation to the translational context of the host genome. This could also explain why the base composition may differ among regions of TEs. For instance, the ORF1 of *LINE*s has usually a less biased base composition than ORF2, possibly because ORF1p needs to be produced in a much larger amount than ORF2p for successful transposition [73,74]. Another source of selection is that the high AT content is a means of escaping repression by the host. TE expression is regulated by methylation of cytosine at CpG, and a low GC content could constitute a defense of TEs against such inactivating mechanisms. The general under-representation of the CpG dinucleotide in TEs is consistent with this possibility [15,110].

## 6. Conclusions

Understanding the causes of the unusual base composition of TEs and the respective role of mutational bias and selective pressure remains an understudied aspect of TE biology. It can however teach us a lot about the nature of the interactions between TEs and their hosts. The long-term persistence of a suboptimal base composition could support a model of coexistence between TEs and host, whereby elements evolve towards a reduced transposition rate that minimizes the negative impact they can have on their host. It has been proposed that such model may be more prevalent than the arms-race model that has prevailed until recently [111], yet additional studies will be necessary to confirm that the biased composition of TEs is in fact adaptive. A second unexplored aspect of TE biology that could be related to base composition is how different TEs coexist and possibly compete in their genomic environment. By analogy with the field of ecology, it has been proposed that TEs are comparable with organisms that are sharing a genomic habitat within which they interact [112,113]. The coexistence within the same genomes of TEs with different base composition could inform on the nature of these interactions. For instance, it is possible that elements with different GC content do not use the same resources (either tRNA or amino acids) and could thus coexist since they are not using the same “genomic niche”.

A better understanding of the compositional bias will require further studies. In particular, comparing the timing and intensity of expression of TEs that differ in base composition, within the same genome, could potentially inform on the functionality and possible adaptive value of a biased base composition. Experimental approaches that synthetically modify the base composition of elements by optimizing or de-optimizing codon usage could also prove informative. Finally, we should not underestimate how biased in favor of model organisms our knowledge of TEs is. Recent investigations on unicellular organisms [70] for instance have challenged the common belief that all TEs are AT rich, and we can expect that studies on more non-model organisms will also bring their share of surprises.

## Data Availability

Not applicable.
